# Comparison of Delta Total Nucleated Cells Assessed via Sysmex XT-2000iV and Sysmex XN-1000V in Effusions in Cats

**DOI:** 10.3390/ani16030366

**Published:** 2026-01-23

**Authors:** Manuela Zanetti, Sara Meazzi, Saverio Paltrinieri, Giulia Mangiagalli, Sara Novellini, Silvia Rossi, Stefanie Klenner-Gastreich, Stefania Lauzi, Alessia Giordano

**Affiliations:** 1 Antech Diagnostics, Mars Petcare Science & Diagnostics, 20129 Milan, Italy; manuela.zanetti@antechdx.com (M.Z.); stefanie.klenner@antechdx.com (S.K.-G.); 2 Department of Veterinary Medicine and Animal Sciences, University of Milan, 26900 Lodi, Italy; sara.meazzi@i-vet.it (S.M.); saverio.paltrinieri@unimi.it (S.P.); sara.novellini28@gmail.com (S.N.); stefania.lauzi@unimi.it (S.L.); 3 I-VET Diagnostics s.r.l., 25020 Flero, Italy; 4 Laboratorio di Analisi Veterinarie MYLAV s.r.l., 20017 Passirana di Rho, Italy; giulia.mangiagalli90@gmail.com; 5 CDVet Veterinary Diagnostic Laboratory, 00195 Rome, Italy; silviarossivet@gmail.com

**Keywords:** cats, cavitary effusions, Delta-FIP, Sysmex XN-1000V

## Abstract

The ratio of the nucleated cell counts (ΔTNC) provided by the Sysmex XT-2000iV DIFF and BASO channels has been proven to be useful in differentiating effusions in cats; in particular, a ΔTNC > 2.5 was found to have high accuracy for effusive FIP. The objective of this study was to compare the ΔTNC obtained with the Sysmex XT-2000iV and Sysmex XN-1000V on different types of effusions. Fresh effusions from cats were evaluated with both analyzers. WBC-BASO, WBC-DIFF, and ΔTNC-XT from the Sysmex XT-2000iV and TNC-WNR, TNC-WDF, and ΔTNC-XN from the Sysmex XN-1000V were recorded and compared. Forty-five feline effusions were collected. The results were not significantly different for all parameters evaluated. The Passing and Bablok regression analysis for ΔTNC-XT and ΔTNC-XN revealed a slope of 1.612 (95% CI 1.030 to 2.316) and an intercept of −0.744 (95% CI −1.591 to −0.120); the Bland–Altman difference plot showed a positive bias of 4.382 (*p* = 0.02). Despite the finding of a positive proportional and constant bias between ΔTNC-XT and ΔTNC-XN, evident especially at high ΔTNC values, samples were classified accordingly between the two analyzers when ΔTNC-XT cut-offs were used, allowing the same accuracy for the identification of suspected and compatible FIP samples.

## Simple Summary

Laboratory evaluation of effusions in cats is mandatory to correctly classify them and as an aid for clinical diagnosis. An important tool is the evaluation of the difference (Delta) in the total nucleated cell count measured by the different channels of an automated hematology analyzer (Sysmex XT-2000iV), which can be particularly helpful for feline infectious peritonitis diagnosis. In this study, we compared the Delta values obtained with the new analyzer Sysmex XN-1000V to the ones obtained with the Sysmex XT-2000iV in effusions from cats with different pathologies. Despite a positive proportional and constant bias, similar accuracy for the classification of samples was found. Similar information may therefore be obtained with the use of the new analyzer in place of the old one.

## 1. Introduction

The laboratory evaluation of effusions includes the assessment of cellularity, necessary for their correct classification [1,2,3], and is commonly performed using automated hematology analyzers. According to the analyzer used, different parameters can be obtained, including the nucleated cell count and the hematocrit. In the Sysmex XN-1000V analyzer (Sysmex Europe GmbH, Norderstedt, Germany), two channels providing a total nucleated cell count (TNC) are present when the “whole blood” method is used, each using two different analytical principles. The white cell differential by fluorescence (WDF) channel counts and differentiates nucleated cells based on the cytoplasmic complexity (side scatter) and fluorescence intensity after the staining of nucleic acids with polymethine dye. This method is analogous to the one used by the Sysmex XT-2000iV (Sysmex Europe GmbH, Norderstedt, Germany) in the DIFF channel. Furthermore, the Sysmex XN-1000V white count and nucleated red blood cell (WNR) channel uses a similar method to the Sysmex XT-2000iV BASO channel, which identifies cells based on volume (forward scatter) and complexity (side scatter) using an acidic reagent that condenses all nucleated cells except basophils (as per the manufacturer’s information sheet). Differently from BASO, the WNR channel partitions cells using side scatter and an additional fluorescent dye that binds nucleic acids and allows the determination of the nucleated red blood cell count [4]. The Delta total nucleated cell count (ΔTNC) is the ratio between the nucleated cell counts provided by the two channels, and both analyzers are able to calculate it. If the cell count is similar in the two channels, as usually occurs when blood is analyzed, the ΔTNC is close to 1. It has been previously described that the Sysmex XT-2000iV ΔTNC (ΔTNC-XT) is a useful parameter when evaluating feline effusions, as it may help to discriminate between effusions due to feline infectious peritonitis (FIP) and effusions due to other etiologies [5]. Specificity (Sp) of 90% for a cut-off of 1.7 and Sp of 100% for a cut-off of 2.5 (with sensitivity of 90% in both cases) for the diagnosis of FIP have been previously reported for ΔTNC-XT [5]. The reason for the high ΔTNC-XT has been identified as the high protein content of FIP effusions, typically ≥3.5 g/dL [6,7], and is mainly due to the presence of large amounts of globulins, as well as fibrinogen [8]. The reagents used in the BASO channel are acidic, leading to possible clot formation; TNC-BASO is therefore usually lower than TNC-DIFF in FIP effusions, leading to a ΔTNC >1 [5,8]. Moreover, Lopes et al. [9] recently suggested the use of ΔTNC with the Sysmex XN-1000V (ΔTNC-XN) as part of an FIP effusion diagnostic approach; according to the authors, the use of the FIP Effusion Index can improve the specificity for FIP diagnosis. The index is calculated by dividing ΔTNC-XN by the albumin/globulin ratio, and a value ≥7.54 is considered diagnostic of FIP.

Compared to the other tests that may provide diagnostic information for FIP [10,11,12,13,14,15], the main advantages of ΔTNC are its ease of use, rapidity, and relatively low cost.

The older instrument has been disused worldwide, possibly being replaced by the latest version; there is hence a need to assess whether the two analyzers give the same information about feline effusions. However, despite the encouraging results reported by Lopes et al. [9], and although the performance of the Sysmex XN-1000V has already been compared with that of the Sysmex XT-2000iV in blood [16,17], information regarding the comparison of the two instruments in the measurement of ΔTNC in effusions is lacking.

The aim of this study was therefore to compare the ΔTNC values obtained with the Sysmex XT-2000iV and XN-1000V analyzers on fresh effusions in cats. Given the similarities between the two analyzers, our hypothesis was that ΔTNC-XN would be similar in magnitude and would give the same information for the diagnosis of FIP compared to ΔTNC-XT.

## 2. Materials and Methods

### 2.1. Study Design and Sample Processing

Effusion samples from cats were processed for routine diagnostic purposes in two major sites at a distance of 40 km from each other: the Department of Veterinary Medicine and Animal Science (DIVAS, Università degli Studi di Milano, Lodi, Italy), defined as site 1, and Biessea Laboratorio di Analisi Veterinarie, an Antech Diagnostics Company (Mars Petcare Science & Diagnostics, Milan, Italy), defined as site 2. An aliquot of each sample was then prepared and delivered to the other site so that the analysis could be performed with both instruments. Some of the samples were collected in other facilities for diagnostic purposes and then aliquoted, delivered, and processed at sites 1 and 2.

Inclusion criteria were defined as follows:

- Peritoneal, pleural, or pericardial feline effusions collected only in EDTA tubes, irrespective of the underlying clinical suspicion;
- Availability of at least 0.5 mL of leftover sample volume from routine analysis;
- Availability of data concerning effusion protein content estimated by refractometric analysis and cytological description.

Additional information collected when available but not considered mandatory included ancillary tests performed on effusions, such as RT-qPCR for feline coronavirus (FCoV); bacterial culture; and biochemical analyses, including cholesterol and triglyceride concentrations.

Exclusion criteria were defined as follows:

- Evident macroscopic flocculation of the sample;
- Leftover volume < 0.5 mL;
- Failure to process the sample at both sites within 48 h of collection.

Samples were analyzed with both the Sysmex XN-1000V (located at site 1) and XT-2000iV (located at site 2) (both instruments from Sysmex Corporation, Kobe, Japan). Samples were processed for routine analysis immediately upon arrival in the lab; for all samples, a 0.5 mL aliquot was placed in a tube (Eppendorf, Hamburg, Germany) and kept refrigerated at 4 °C. Samples were then processed within 24 h in the other lab, so that the count could be performed with both analyzers. Samples collected in facilities other than sites 1 and 2 were divided into two 0.5 mL aliquots and delivered refrigerated to both labs within 12–24 h.

Upon arrival, samples were left at room temperature and gently mixed for 15 min before performing the analysis.

### 2.2. Instrumental Analysis

Before starting the analysis, both instruments were checked on a daily basis using a two-level commercial quality control material (QCM) and a three-level quality control material for the XT-2000iV and XN-1000V, respectively. All QC material was provided by the manufacturer (Sysmex Europe GmbH, Norderstedt, Germany).

For each analyzed sample, the following parameters were recorded: TNC-BASO, TNC-DIFF (for simplicity, named WBC-BASO and WBC-DIFF), and ΔTNC-XT generated by the XT-2000iV; TNC-WNR, TNC-WDF, and ΔTNC-XN generated by the XN-1000V.

### 2.3. Statistical Analysis

Statistical analysis was conducted using the Analyse-it software (version 6.15.4) for Microsoft Excel (Analyse-it Software Ltd., Leeds, UK), with the significance level set at *p* < 0.05.

ΔTNC-XT and ΔTNC-XN were compared to each other using a non-parametric *t* test for paired data (Wilcoxon signed rank test). The same test was used to compare both the WBC-BASO values with the TNC-WNR values and the WBC-DIFF values with the TNC-WDF values. These tests were run either on the whole dataset or on samples grouped according to the site at which they were first analyzed.

For ΔTNC-XT vs. ΔTNC-XN, WBC-BASO vs. TNC-WNR, and WBC-DIFF vs. TNC-WDF values, the agreement between the results obtained with the two analyzers was evaluated using Passing and Bablok regression analysis and a Bland–Altman difference plot.

The concordance between ΔTNC-XT and ΔTNC-XN in identifying values higher than the two published cut-offs of 1.7 and 2.5, used to identify suggestive and compatible FIP samples, respectively, was assessed using Cohen’s kappa test. To evaluate the concordance in identifying samples suggestive of FIP (cut-off >1.7), all samples with ΔTNC-XT and ΔTNC-XN > 1.7 were considered positive, and all samples with ΔTNC-XT and ΔTNC-XN < 1.7 were considered negative. The same dichotomization was performed to consider the concordance in identifying samples compatible with FIP (cut-off for positivity: >2.5). The level of agreement was assessed as follows: “absent” for k values between 0.00 and 0.20; “weak” for k values between 0.21 and 0.40; “moderate” for k values between 0.41 and 0.60; “good” for k values between 0.61 and 0.80; and “almost perfect” for k values greater than or equal to 0.81 [5].

## 3. Results

### 3.1. Study Population and Description of Samples

A total of 45 samples matched all inclusion criteria and were included in the study. In particular, 11/45 were collected at site 1 and 20/45 at site 2, and 14/45 were collected in other facilities. Demographic information is available in the Supplementary Materials (Table S1).

According to their macroscopic appearance, protein content, total nucleated cell count, cytological evaluation, and other ancillary tests, samples were classified as follows: suspected FIP (*n* = 14), non-septic exudate (*n* = 12), protein-rich transudate (*n* = 4), neoplastic effusion (*n* = 6), chylous effusion (*n* = 4), protein-poor transudate (*n* = 3), uroperitoneum (*n* = 1), and septic effusion (*n* = 1) [18,19]. Of the samples classified as suspected FIP, FCoV RT-qPCR on effusion was available in seven cases, and it was positive in 6/7 cases.

### 3.2. Comparison Between ΔTNC-XT and ΔTNC-XN

A preliminary statistical comparison did not reveal differences in samples processed first at site 1 or at site 2 or simultaneously; therefore, the comparison of the results obtained with the two instruments was performed on the whole dataset.

**Figure 1:**
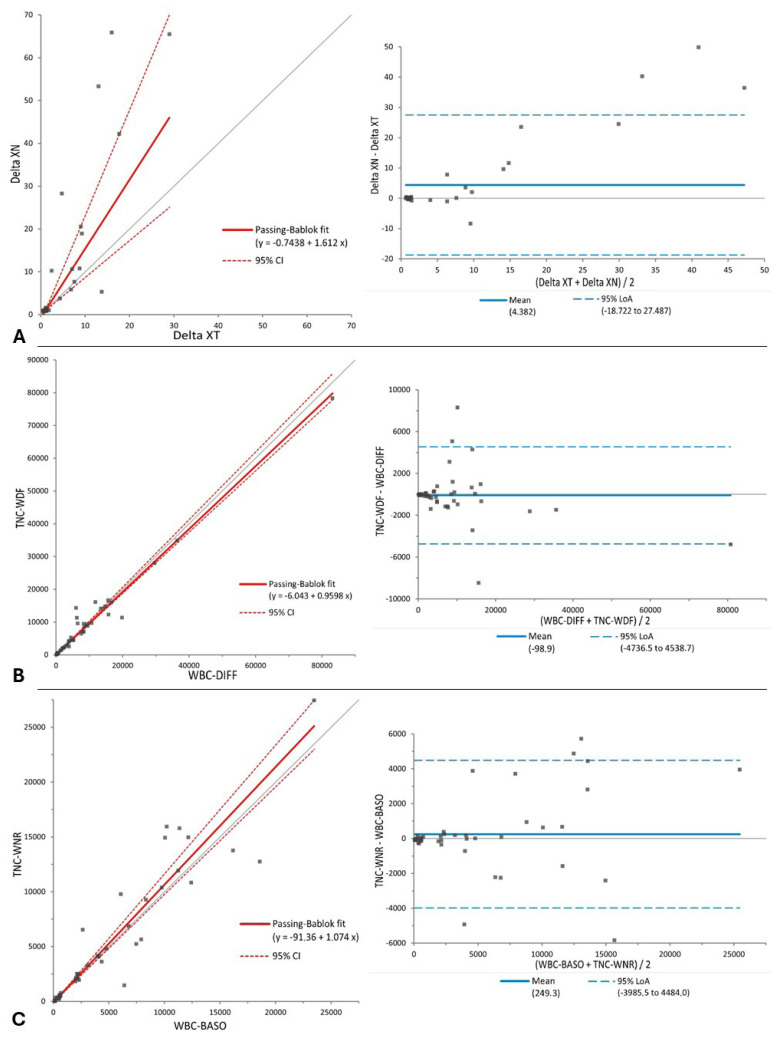
Passing–Bablok regression analysis (**left**) and Bland–Altman difference plot (**right**) for (**A**) ΔTNC values of effusions measured with Sysmex XT-2000iV (Delta-XT) and Sysmex XN-1000V (Delta-XN); (**B**) total nucleated cell count measured with Sysmex XT-2000iV DIFF channel (WBC-DIFF) and with Sysmex XN-1000V WDF channel (TNC-WDF); (**C**) total nucleated cell count measured with Sysmex XT-2000iV BASO channel (WBC-BASO) and with Sysmex XN-1000V WNR channel (TNC-WNR).

No significant differences were found between ΔTNC-XT and ΔTNC-XN, WBC-BASO and TNC-WNR, or WBC-DIFF and TNC-WDF (Table 1). Both a constant and proportional error, along with a significant bias (*p* = 0.02), were found between ΔTNC-XT and ΔTNC-XN. A proportional error was found between WBC-BASO and TNC-WNR, but no constant error or significant bias was observed; no constant or proportional errors or significant bias were found in the comparison between WBC-DIFF and TNC-WDF (Table 1, Figure 1).

### 3.3. Concordance Between Sysmex XN-1000V and Sysmex XT-2000iV in Identifying Samples with High ΔTNC-XT

In 30/45 cases, the ΔTNC-XT value was <1.7, while, in 15/45 cases, it was >1.7 (Table 2); among the latter, 13/15 cases showed a ΔTNC-XT >2.5. ΔTNC-XN was <1.7 in 31/45 cases; in 14/45 cases, ΔTNC-XN was both >1.7 and >2.5. The 30 samples with both ΔTNC-XT and ΔTNC-XN <1.7 included non-septic exudates (11/30), neoplastic effusions (6/30), protein-rich transudates (4/30), chylous effusions (4/30), protein-poor transudates (2/30), suspected FIP (2/30), and uroperitoneum (1/30). The 14 samples with both ΔTNC-XT and ΔTNC-XN > 1.7 included suspected FIP (13/14) and a septic effusion (1/14). The 12 samples with both ΔTNC-XT and ΔTNC-XN > 2.5 included suspected FIP (11/12) and a septic effusion (1/12). Overall, the concordance between the methods in classifying samples with a ΔTNC-XT < 1.7 was almost perfect (k = 0.949; 95% CI: 0.851–1.000).

The only discordant sample had a ΔTNC-XT value of 1.85 and a ΔTNC-XN value of 1.03 and it was classified as a protein-poor transudate. The ΔTNC-XN correctly identified all samples with ΔTNC-XT > 2.5 and <2.5 except one, which had a ΔTNC-XT result of 2.45 and a ΔTNC-XN result of 10. The concordance was again considered almost perfect in classifying samples with a ΔTNC > 2.5 (k = 0.947; 95%CI: 0.845–1.000) (Table 3).

## 4. Discussion

The results of this study demonstrate comparable findings between the two instruments, despite the presence of a statistically significant difference between ΔTNC-XN and ΔTNC-XT, indicating that ΔTNC-XN values are not strictly interchangeable with those from ΔTNC-XT.

The mechanism responsible for high ΔTNC-XN values is likely the same as reported for ΔTNC-XT [5,15]: the total nucleated cell count obtained with the WNR channel is lower compared to that obtained with the WDF channel in those samples with high protein content, probably due to fibrinogen clotting in the acidic environment and cells’ entrapment.

ΔTNC-XN demonstrates a positive proportional and constant bias compared to ΔTNC-XT. The bias impact is especially evident in those samples with very high ΔTNC values. From a clinical perspective, the results’ interpretation does not change: the concordance analysis demonstrates that “negative” samples (i.e., samples that had ΔTNC-XT < 1.7) or samples with ΔTNC-XT > 2.5 are correctly classified by both analyzers. Still, considering this analytical difference, and the fact that our samples did not have a final diagnosis of FIP, there is likely the need to establish analyzer-specific cut-offs, and we direct the reader to studies that consider the clinical validation of ΔTNC-XN in the diagnosis of FIP [9]. Indeed, it is very likely that, based on the analytical differences, the proportional errors, and the biases mentioned above, the cut-off values derived from ΔTNC-XT cannot be directly extrapolated to ΔTNC-XN without clinical validation and, ideally, prospective evaluation in confirmed FIP cases. As support for this hypothesis, in the study of Lopes et al. [9], the cut-offs established for ΔTNC-XN were higher than those recommended for ΔTNC-XT (ΔTNC-XN > 2.1 is likely consistent with FIP and ΔTNC-XN > 4.9 may confirm a clinical diagnosis of FIP). Unfortunately, also in that study, the diagnosis of FIP was achieved based on an algorithm combining results from different tests (cytology, Rivalta’s test, RT-qPCR, albumin/globulin ratio) in vivo, but no immunohistochemistry or necropsies were performed for the confirmation of the diagnosis.

Interestingly, in our caseload, two samples showed discordant classification between the two analyzers. One was classified as suggestive of FIP with the XT-2000iV (ΔTNC-XT between 1.7 and 2.5) but negative with the XN-1000V (ΔTNC-XN < 1.7). The sample was classified as a protein-poor transudate due to the low total protein concentration and low cellularity. The cytologic evaluation and macroscopic appearance of the specimen were not suggestive of FIP, so it is possible that the higher result obtained with the XT-2000iV was an artifact caused by the reduced analytical sensitivity of the analyzer at very low cell counts, similarly to what was reported in a previous study [5]. The other discordant sample was classified as suggestive of FIP by the XT-2000iV (ΔTNC-XT > 1.7) and consistent with FIP by the XN-1000V (ΔTNC-XN > 2.5). This is not surprising considering the positive proportional bias found between the instruments, and this finding again supports the need for analyzer-specific cut-offs.

Among the limitations of this study, there is the inclusion of effusions irrespective of the clinical suspicion or diagnosis. The concordance of ΔTNC-XT and ΔTNC-XN in classifying effusions as suggestive or consistent with FIP is based on the diagnostic accuracy of ΔTNC-XT, previously established based on ΔTNC-XN. The 14 effusions included in the “suspected FIP” category based on the gross, cytological, and physicochemical features of the samples may or may not have contained FIP, since there was no additional clinical or laboratory information to support this diagnosis. More specifically, of the samples classified as suspected FIP, FCoV RT-qPCR on effusion was available in seven cases, and it was positive in 6/7 cases. Based on this result, as well as on the compatible physicochemical and cytological findings, a diagnosis of FIP was very likely due to the high specificity of such molecular tests [14,18]. Conversely, a negative RT-qPCR result in the other cat does not rule out a diagnosis of FIP, as the sensitivity of RT-qPCR in effusion samples varies considerably, with reported values ranging from 72% to 100%, depending on the study and the methodology used [14,15,18]. However, none of the cats enrolled in this study, including the seven cats on which RT-qPCR was performed, had further results of other clinical–pathological tests that may have increased the probability of FIP (routine hematology, serum protein electrophoresis, measurement of acute-phase proteins) [10]. Therefore, although, based on the physicochemical and cytological analysis of the effusion, the likelihood of FIP in these 14 cats was high, and the study demonstrated analytical concordance between ΔTNC-XN and ΔTNC-XT, our caseload did not include gold-standard-confirmed cases. It was therefore not possible to validate the clinical diagnostic performance of ΔTNC-XN for FIP, for which we refer to other studies. Conversely, the availability of other information on clinical signs or on clinicopathological tests may support the diagnosis of FIP also in some of the cats for which the effusion was classified as a non-septic exudate or a protein-rich effusion. Interestingly, both instruments indicated a septic effusion as suggestive of or consistent with FIP (ΔTNC-XN and ΔTNC-XT > 1.7 and >2.5); this finding is not surprising since septic effusions contain fibrinogen or other high-molecular-weight proteins that may clot in the presence of acidic reagents. Although this has not been recorded in previous studies on ΔTNC-XT [5,15], septic exudates are known to induce positive results with the Rivalta test [19].

The present study was not focused on the assessment of the ability of ΔTNC-XN in confirming or excluding a diagnosis of FIP. To this aim, further studies on whether the clinical and laboratory approach allows one to achieve a final diagnosis of FIP or of diseases causing effusions that mimic FIP are needed. Rather, this study was designed based on the agreement between the ΔTNC values provided by the two instruments, and, from this standpoint, the results may stand alone even in the absence of information about the final diagnosis in the enrolled cats.

Another possible limitation is the collection of samples at multiple locations; although, in the current study, we obtained similar results regardless of the place of first processing and the time that elapsed between analyses with the two instruments, considering the labile nature of effusions’ cytochemical characteristics, a single location would have been preferrable to avoid any possible random error caused by sample transportation, possible sample degradation/alteration during transport, sample handling by multiple laboratory personnel, and a general lack of standardization in sample processing.

## 5. Conclusions

In conclusion, despite the methodological differences between the WNR channel of the Sysmex XN-1000V and the BASO channel of the XT-2000iV, ΔTNC-XN remains a reliable parameter for the evaluation of feline effusions. The strong analytical concordance between ΔTNC-XN and ΔTNC-XT demonstrates that both analyzers yield comparable results, and the observed proportional bias is unlikely to have clinical significance. Consequently, ΔTNC-XN can be confidently adopted in diagnostic laboratories for the evaluation of feline effusions and the presumptive identification of FIP, provided that analyzer-specific diagnostic thresholds are established.

## Figures and Tables

**Table 1:** Data regarding comparison and agreement of the paired parameters. Values are expressed as absolute numbers. CI: confidence interval. Significance is set at *p* < 0.05.

	ΔTNC-XT vs. ΔTNC-XN	WBC-BASO vs. WNR	WBC-DIFF vs. WDF
Statistical difference (*p* value)	0.615	0.196	0.765
Slope (95% CI)	1.612 (1.030 to 2.316)	1.074 (0.984 to 1.171)	0.960 (0.935 to 1.043)
Intercept (95% CI)	−0.744 (−1.591 to −0.120)	−91.36 (−200.9 to −29.1)	−6.04 (−73.97 to 39.04)
Bias (*p* value)	4.382 (0.016)	249.3 (0.443)	−98.9 (0.780)

**Table 2:** Contingency table showing the distribution of results regarding ΔTNC obtained with the Sysmex XT (ΔTNC-XT) and XN (ΔTNC-XN) using the threshold of 1.7, considered as suggestive of FIP in a previous study [12].

	ΔTNC-XT < 1.7	ΔTNC-XT > 1.7	Total Samples ΔTNC-XN
ΔTNC-XN < 1.7	30	1	31
ΔTNC-XN > 1.7	0	14	14
Total Samples ΔTNC-XT	30	15	

**Table 3:** Contingency table showing the distribution of results regarding ΔTNC obtained with the Sysmex XT (ΔTNC-XT) and XN (ΔTNC-XN) using the threshold of 2.5, considered as consistent with FIP in a previous study [12].

	ΔTNC-XT < 2.5	ΔTNC-XT > 2.5	Total Samples ΔTNC-XN
ΔTNC-XN < 2.5	31	0	31
ΔTNC-XN > 2.5	1	13	14
Total Samples ΔTNC-XT	32	13	

## Data Availability

The raw data supporting the conclusions of this article will be made available by the authors on request.

## Supplementary Materials

The following supporting information can be downloaded at https://www.mdpi.com/article/10.3390/ani16030366/s1: Table S1: Demographic information of the samples, including sex, age and breed. Age is expressed in years. F: female; SF: spayed female; M: male; CM: castrated male; U: unknown; DSH: domestic shorthair.

## Abbreviations

The following abbreviations are used in this manuscript:

ΔTNC	Delta total nucleated cell count
FCoV	Feline coronavirus
FIP	Feline infectious peritonitis
LR	Likelihood ratio
QCM	Quality control material
RT-qPCR	Real-time reverse transcriptase polymerase chain reaction
Sp	Specificity
TNC	Total nucleated cell count
WDF	White cell differential channel by fluorescence
WNR	White and nucleated red blood cell count
