# Supplementary material for: Comparison of Delta Total Nucleated Cells Assessed via Sysmex XT-2000iV and Sysmex XN-1000V in Effusions in Cats
Source: Animals (Basel). 2026 Jan 23;16(3):366. doi: 10.3390/ani16030366 (PMC12896741; doi:10.3390/ani16030366)
Supplement: Supplementary file 1 [file animals-16-00366-s001.zip › animals-4078913-supplementary.pdf]

Supplemental Table S1. Demographic information of the samples, including sex, age and breed. Age is expressed in years. F: female; SF: spayed female; M: male; CM: castrated male; U: unknown; DSH: domestic shorthair.

Sample n°	Sex	Age (years)	Breed
1	F	0,8	DSH
2	M	2	Persian
3	M	0,9	DSH
4	SF	5	DSH
5	M	1	Siamese
6	SF	19	DSH
7	M	18	U
8	F	2	DSH
9	F	2	DSH
10	CM	5	DSH
11	F	8	U
12	M	10	DSH
13	M	11	DSH
14	F	2	DSH
15	F	18	DSH
16	SF	12	DSH
17	M	3	DSH
18	M	10	DSH
19	M	12	DSH
20	M	10	DSH
21	F	10	DSH
22	M	2	DSH
23	F	13	DSH
24	F	0,6	DSH
25	CM	11	DSH
26	M	3	DSH
27	M	1	DSH
28	M	14	DSH
29	F	0,4	DSH
30	SF	10	DSH
31	F	0,9	DSH
32	CM	11	DSH
33	F	15	DSH
34	U	5	Persian
35	CM	8	DSH
36	F	1	British
37	M	15	DSH
38	M	10	DSH
39	F	10	DSH
40	M	3	Siberian
41	U	U	DSH
42	SF	12	DSH
43	F	12	DSH
44	F	16	DSH
45	F	0,4	DSH
